# OVERCOMING BARRIERS IN DETECTING SUBJECTIVE COGNITIVE DECLINE: A QUALITATIVE STUDY WITH HEALTH CARE PROFESSIONALS

**DOI:** 10.1093/geroni/igae098.1383

**Published:** 2024-12-31

**Authors:** Ryan Mace, Joshua Cohen, Christine Ritchie, Olivia Okereke, Stephen Bartels, Bettina Hoeppner, Judson Brewer, Ana-Maria Vranceanu

**Affiliations:** Massachusetts General Hospital/Harvard Medical School, Boston, Massachusetts, United States; Massachusetts General Hospital, Boston, Massachusetts, United States; Mass General Hospital and Harvard Medical School, Boston, Massachusetts, United States; Massachusetts General Hospital, Boston, Massachusetts, United States; Massachusetts General Hospital/Harvard Medical School, Boston, Massachusetts, United States; Massachusetts General Hospital/Harvard Medical School, Boston, Massachusetts, United States; Brown University Medical School/Brown University School of Public Health, Providence, Rhode Island, United States; Massachusetts General Hospital, Boston, Massachusetts, United States

## Abstract

Identifying older adults with subjective cognitive decline (SCD) is imperative for early interventions targeting modifiable risk factors for Alzheimer’s Disease and Related Dementias (ADRD). However, with fewer than half of older patients with SCD engaging in discussions about their concerns with healthcare professionals, a significant gap in current practice is evident. To address this gap, we engaged healthcare professionals (physicians and medical staff) in a qualitative study that aimed to: 1) understand barriers and facilitators to identifying SCD, and 2) develop strategies to guide the implementation of screening and assessment tools into routine care. We conducted live video focus groups with 26 healthcare professionals (Mean experience = 17 ± 13.6 years) from 5 clinics that treat older patients (e.g., psychiatry, neurology, integrative medicine, memory care). We analyzed the qualitative data using a hybrid deductive–inductive approach. We proposed concrete strategies for addressing barriers to SCD identification using the Expert Recommendations for Implementing Change (ERIC) framework. Four themes on barriers to SCD identification emerged: 1) the clinical heterogeneity of SCD, 2) variability in patients’ self-identification, 3) variability in clinical assessment practices, and 4) need for additional research and standardization. ERIC-informed strategies to address barriers included: education and training for providers, standardized diagnostic protocols, incorporating caregiver reports, and promoting de-stigmatizing language. Our study contributes to the symposium theme by delineating the intricacies of identifying SCD among older adults and by proposing ERIC framework-based strategies that align with the need for novel screening and assessment tools.

